# Mesenchymal stem cells are mobilized from the bone marrow during inflammation

**DOI:** 10.3389/fimmu.2013.00049

**Published:** 2013-03-04

**Authors:** Jasper J. Koning, Gijs Kooij, Helga E. de Vries, Martijn A. Nolte, Reina E. Mebius

**Affiliations:** ^1^Department of Molecular Cell Biology and Immunology, VU University Medical CenterAmsterdam, Netherlands; ^2^Department of Hematopoiesis, Adaptive Immunity Lab, Sanquin Research and Landsteiner Laboratory AMC/UvAAmsterdam, Netherlands

**Keywords:** MSC mobilization, experimental allergic encephalomyelitis, inflammation and MSC, bone marrow and MSC, MSC and IFNg, CD70

## Abstract

Mesenchymal stem cells (MSCs) show great therapeutic potential for the treatment of various immune mediated diseases, including Multiple Sclerosis (MS). Systemic administration of MSCs during experimental allergic encephalomyelitis (EAE), an animal model for MS, was shown to reduce the infiltration of T cells, B cells, and macrophages into the CNS. Whether endogenous MSCs are mobilized and potentially modulate the severity of disease is not known. Here we show that during the acute phase of EAE, MSCs numbers in the bone marrow were severely reduced, which restored to control levels during the progressive phase of the disease. The number of bone marrow MSCs inversely correlated with the number of both CD4 and CD8 T cells present in the bone marrow indicating a link between activated T cells and MSC mobilization. Analysis of CD70-transgenic mice, which have a constitutively activated immune system and elevated number of activated T cells in the bone marrow, showed severely reduced number of bone marrow MSCs. Transfer of T cells that were activated through their CD27 receptor reduced the number of bone marrow MSCs dependent on IFN-y. These data provide a mechanism by which MSCs can be mobilized from the bone marrow in order to contribute to tissue repair at a distant location.

## Introduction

Mesenchymal stem cells (MSCs) are described as multipotent stromal cells, mainly residing in the bone marrow, although they can also be found in low numbers in almost all adult tissues (Kuznetsov et al., 2001; da Silva et al., 2006). MSCs can differentiate into various cells of the mesodermal lineage which include osteoblasts, adipocytes, and chondrocytes as well as myelosupportive stroma which, together with endothelial cells, are all constituents of the heterogeneous bone marrow stroma (Friedenstein et al., 1966; Kuznetsov et al., 1997; Prockop, 1997; Pittenger et al., 1999; Tokoyoda et al., 2010). Stromal cells within the bone marrow support the maintenance of hematopoietic stem cells (HSCs) (Mendez-Ferrer et al., 2010).

In the bone marrow, MSCs were identified as a population of nestin-expressing cells which exclusively contained colony-forming-unit fibroblast (CFU-F) activity and depletion of nestin^pos^ cells reduced the HSC-activity in bone marrow indicating that these cells are HSC-niche components (Mendez-Ferrer et al., 2010). However, whereas HSCs crucially depend on stem cell factor (SCF) produced by niche cells, deletion of SCF from nestin^pos^ cells did not affect HSC frequency in the bone marrow (Ding et al., 2012), suggesting that other cell types also contribute to HSC maintenance through SCF production. Indeed, both endothelial as well as leptin receptor-expressing perivascular cells were identified as the source of SCF and deletion of either population clearly reduced HSCs numbers in bone marrow (Ding et al., 2012). Therefore, multiple cell types within the heterogeneous bone marrow stroma support HSC-niche maintenance under homeostatic conditions.

In analogy to the supportive function of lymph node stromal cells in immune cell homeostasis (Koning and Mebius, 2011), bone marrow stromal cells can also support maintenance of various lymphocytes (Tokoyoda et al., 2010). The lack of distinctive structures such as lymphatic endothelial cells as well as specialized high endothelial venules in the bone marrow however, may reflect differences in the functional role of these organs in lymphocyte homeostasis. Verily, whereas lymph node stromal cells are involved in the initial phase of immune responses, bone marrow stromal cells participate in maintaining memory cells, both plasma cells and CD4^pos^ T cells (Tokoyoda et al., 2010). Memory T cells reside in contact with VCAM-1^pos^ stromal cells that express IL-7, a T cell survival cytokine (Fry and Mackall, 2005; Tokoyoda et al., 2009). Bone marrow plasma cells locate near CXCL12 abundant reticular (CAR) cells, while the preponderance of HSCs also locates near CAR cells. Therefore, whereas in lymph nodes specialized micro-environmental stromal subsets, which support specific types of immune cells, have been described (Koning and Mebius, 2011), such subdivisions for bone marrow stromal cells are less well defined. Nevertheless, the overlapping supportive and suppressive roles of stromal cell microenvironments in lymphocyte homeostasis and function, respectively, are shared by all stromal cells despite distinct anatomical locations (Jones et al., 2007). MSCs are precursors for stromal cells in various organs, and they share immunosuppressive effects with stromal cells. Therefore, MSCs are thought to serve as good candidates to treat immune mediated diseases including Multiple Sclerosis (MS) (Uccelli et al., 2008).

MS is a chronic inflammatory disease resulting in demyelination and axonal loss throughout the central nervous system (CNS), with unknown cause and only limited treatment options (Ewing and Bernard, 1998; Noseworthy et al., 2000; Lassmann et al., 2001). Spontaneous remyelination that contributes to functional recovery is limited resulting in a relentless increase in disability during disease progression (Scolding and Franklin, 1998; Bruck et al., 2003; Fancy et al., 2010).

The effectiveness of administration of MSCs for treatment of MS patients needs to be explored in more detail, but animal studies in rodents show promising perspectives for future treatments. The most commonly used animal model for MS research is murine experimental allergic encephalomyelitis (EAE). This model resembles both the inflammatory phase, i.e., the generation of autoreactive myelin specific T cells, as well as the neurodegenerative phase of the disease, i.e., destruction of the myelin sheath around the axons and subsequent loss of axons, as observed in human disease (Steinman, 2001). Several studies have shown that systemic administration of MSCs at disease onset ameliorated EAE and decreased infiltration of T cells, B cells and macrophages into the CNS (Zappia et al., 2005; Kassis et al., 2008). In these studies MSCs exerted immunomodulatory effects via inhibition of T cell activation and proliferation.

A recent study indicates that MSCs also harbor direct neuroprotective effects. It was demonstrated that administration of MSCs at the onset of EAE remarkably reduced the levels and activity of anti-oxidant molecules *in vivo* (Lanza et al., 2009). Subsequently, using an *in vitro* model system, the authors showed that upon induction of oxidative stress within a neuroblastoma celline, MSC-conditioned medium suppressed the upregulation of anti-oxidant molecules indicating a direct neuroprotective effect of MSCs (Lanza et al., 2009).

While it was shown that MSCs migrate to the brain upon *i.v*. administration after the experimental induction of stroke, only a small percentage of these cells enters the CNS parenchyma (Eglitis et al., 1999). And although MSCs were reported to transdifferentiate *in vitro* into neural cells (Kopen et al., 1999) most studies so far indicate that MSCs do not transdifferentiate *in vivo* during EAE, despite their presence in spinal cord (SPC) and brain after systemic administration (Zappia et al., 2005; Gerdoni et al., 2007). Therefore, the positive effect of MSC administration on the disease course of EAE is mostly through modulation of immune cells although direct neuroprotective effects may also play a role.

All studies which addressed a potential therapeutic effect of MSCs on EAE disease outcome focused on administration of exogenous MSCs (Zappia et al., 2005; Gerdoni et al., 2007; Kassis et al., 2008; Lanza et al., 2009). However, so far there is no data concerning the behavior of endogenous MSCs during the course of EAE. Since the bone marrow is the major source of MSCs, we investigated the presence of bone marrow MSCs during the course of MOG induced EAE. We found severely reduced numbers of bone marrow MSCs at the peak of disease, which restored to control levels upon progression into the chronic phase. Activated CD4 T cells in the CNS, which produce pro-inflammatory molecules such as IFN-y, TNF-α, IL-17, lymphotoxin, and GM-CSF, are considered to play a central role in the pathogenesis of MS and EAE (Zamvil and Steinman, 1990; Sospedra and Martin, 2005; Segal, 2010; Codarri et al., 2011). Analysis of the immune cells within the bone marrow revealed a significant negative correlation between CD4^pos^ and CD8^pos^ T cells and MSC, such that high numbers of either T cell subset coincided with low numbers of bone marrow MSCs, suggesting a T cell mediated effect on MSC mobilization. Analysis of MSC numbers in the bone marrow of mice with constitutively activated T cells showed a strong reduction of MSCs in the bone marrow. Indeed, transfer of T cells, which were subsequently activated through their CD27 receptor, demonstrates a role for T cells in reducing the number of MSCs. While prolonged production of IFN-y in the bone marrow seemed to reduce MSC numbers, short term mobilization by T cells was independent of T cell derived IFN-y.

## Results

### Reduced number of mesenchymal stem cells is present in the bone marrow during EAE

**Figure 1:**
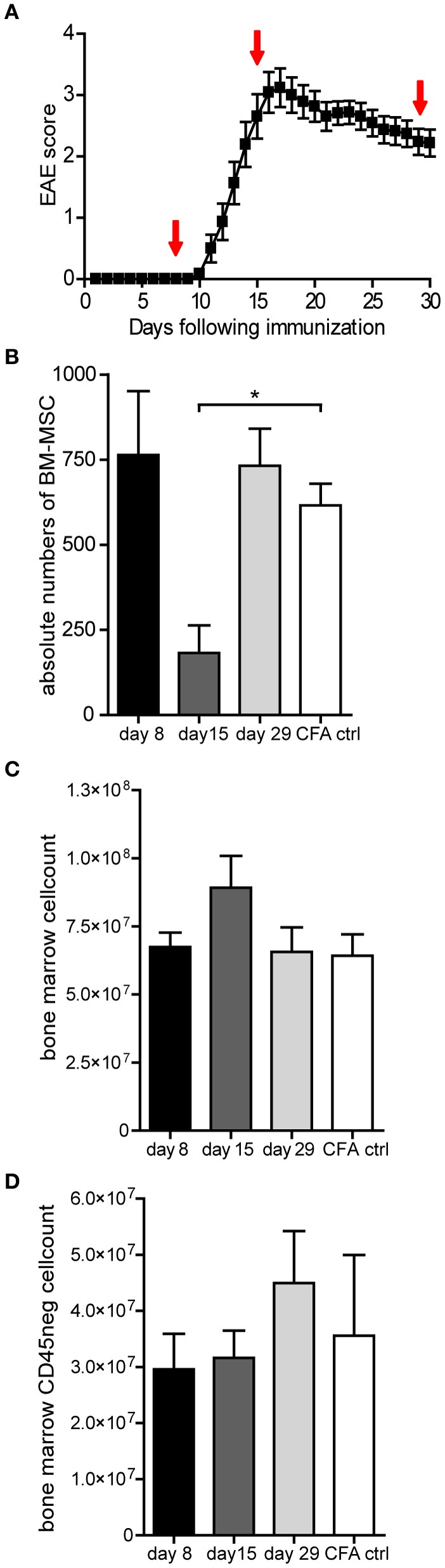
**The number of MSCs decreases transiently in the bone marrow during EAE. (A)** Clinical signs of rMOG (1–125) induced EAE showing mean clinical scores (± SEM). Mice were examined daily for clinical signs of EAE and were scored as followed: 0, no disease; 1, limp tail; 2, hind limb weakness; 3, complete hind limb paralysis; 4, hind limb paralysis plus forelimb paralysis; and 5, moribund or dead. Bone marrow was isolated at day 8, 15, and 29 after immunization as indicated by arrows (*n* = 3 per group). **(B)** Numbers of MSCs present in the bone marrow during the course of EAE analyzed at the indicated timepoints after EAE induction. Total numbers of MSCs were determined by colony forming unit (CFU) assay and numbers of CFUs are expressed as mean ± SEM (*n* = 3 or more per group, one way ANOVA, ^*^p < 0.05). **(C,D)** Bone marrow cell counts and total number of CD45^neg^ cells of EAE mice during the course of EAE analyzed at the indicated timepoints. Data represent mean ± SEM of EAE induced mice (*n* = 3 or more per group).

Over the past years there has been increasing evidence that administration of MSCs decreases the severity of EAE (Zappia et al., 2005; Kassis et al., 2008; Lanza et al., 2009). However, so far no data has been presented concerning the behavior of endogenous bone marrow MSCs during the course of EAE. Therefore, we induced EAE with recombinant myelin oligodendrocyte glycoprotein (rMOG) and analyzed total numbers of MSCs in the bone marrow, the major reservoir for MSCs, at various timepoints after disease induction (day 8, 15, and 29). At day 8 after disease induction, mice are still in the inductive phase and exhibit no clinical signs yet. However, at day 15 after disease induction, mice suffered from severe clinical signs varying from hind leg bending (score 2) to complete hind leg paralysis (score 4) which is accompanied by infiltration of immune cells, such as macrophages as well as T cells, in white matter lesions of the brain (Kooij et al., 2009). During the progressive phase of the disease (day 29), clinical symptoms were slightly improved (Figure 1A).

Total numbers of MSC, present in the bone marrow, were determined using the colony-forming units-fibroblast (CFU-F) assay, which exploits specialized medium to allow selective outgrowth of MSCs. At day 8, shortly before clinical onset of the disease, no change in the amount of bone marrow MSCs could be observed when compared to CFA induced control animals (Figure 1B). Strikingly, at the peak of the disease (day 15), we observed a dramatic reduction in the amount of MSCs present in the bone marrow (Figure 1B). This decrease in bone marrow MSCs appeared to be transient, since numbers were comparable to control mice at the chronic phase (day 29), when clinical symptoms started to improve (Figures 1A,B). In mice that exhibit no clinical symptoms despite disease induction, we could not observe changes in MSC numbers (data not shown).

The decrease in absolute numbers of MSC at day 15 was not the result of a decrease in bone marrow cellularity, since total bone marrow cell counts were comparable to CFA control mice (Figure 1C). Bone marrow MSCs are precursors for bone marrow mesenchymal stromal cells and although it appeared that MSCs were mobilized from the bone marrow, it is conceivable that MSCs have differentiated locally into bone marrow mesenchymal stromal cells, thus remaining within the bone marrow. To address whether the number of bone marrow stromal cells had increased, the total number of CD45^neg^ cells in the bone marrow was determined by FACS analysis at day 8, 15, and 29 after disease induction. These analyses showed no significant changes, suggesting that the stromal compartment did not expand substantially (Figure 1D). These data suggest that MSCs are mobilized from the bone marrow during the course of EAE.

### The number of MSCs negatively correlates with both CD4 and CD8 T cells during EAE

**Figure 2:**
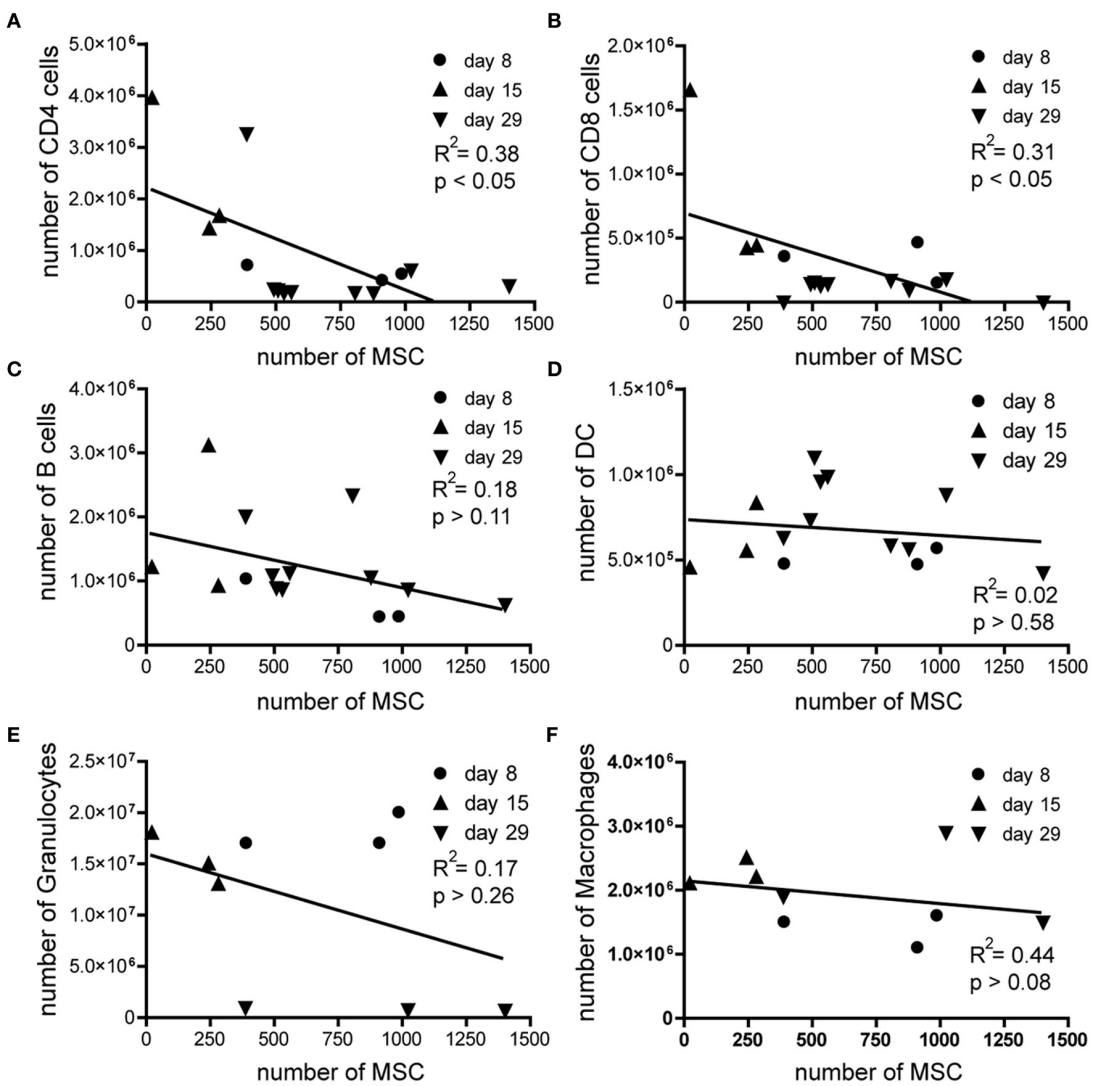
**The number of MSCs negatively correlates with both CD4 and CD8 T cells during EAE.** Absolute numbers of MSCs plotted against the absolute numbers of **(A)** CD4 T cells, **(B)** CD8 T cells, **(C)** B cells, **(D)** Dendritic cells, **(E)** Granulocytes, and **(F)** macrophages during the coarse of EAE. Correlation was analyzed by using linear regression analysis.

Mobilization of MSCs is a multistep process, in which the initial release from their niche is followed by active migration of MSCs across bone marrow endothelium to eventually reach the bloodstream (Pitchford et al., 2009). The factors involved in mobilization of MSCs are not completely elucidated but it has been shown that treatment with VEGF followed by CXCR4-antagonist infusion leads to mobilization of MSCs out of the bone marrow (Pitchford et al., 2009). Recombinant-MOG-induced EAE is primarily a T cell mediated disease (Zamvil and Steinman, 1990), although the disease additionally involves other immune cells (Hemmer et al., 2002). These immune cells may in fact migrate to the bone marrow, where they could mediate the mobilization of MSCs. We therefore analyzed the composition of the immune cells in bone marrow with FACS analysis during the course of the disease and correlated the number of immune cells with the number of MSCs present in the bone marrow. Interestingly we observed a significant negative correlation between the number of both CD4^pos^ and CD8^pos^ T cells and MSCs, such that high number of T cells correlated with low numbers of MSCs (Figures 2A,B), independent of time after induction of the disease. We did not observe such a correlation with B cells, dendritic cells, granulocytes, or macrophages (Figures 2C–F).

**Figure 3:**
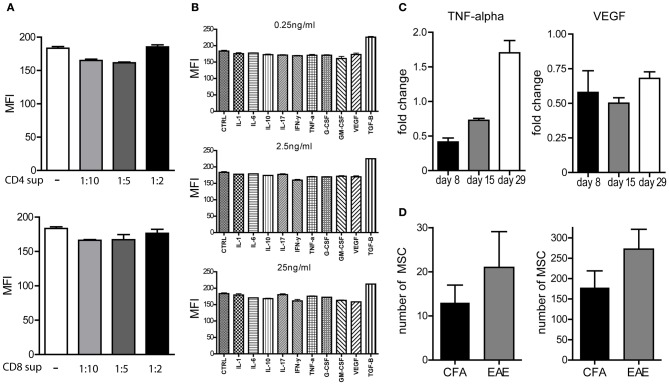
**Proliferation of MSCs not affected upon cytokine treatment. (A,B)** *In vitro* proliferation of CFSE labeled bone marrow derived MSCs from healthy mice in the presence of different concentrations of supernatants from CD3/CD28 beads activated CD4 or CD8 T cells **(A)** or various growth factors or cytokines **(B)** during 72 h measured by MFI of CFSE. Experiments were performed three times in duplo and a representative example is shown. **(C)** TNF-alpha and VEGF-A levels determined by Luminex assay in supernatants of bone marrow samples which were re-stimulated *in vitro* with rMOG for 72 h. Experiments performed in duplo and data represent fold change compared to CFA induced control mice (*n* = 3 per group). **(D)** Absolute numbers of MSCs in cervical lymph nodes and spinal cord in EAE animals at day 29 after immunization (*n* = 3, mean ± SEM).

Therefore, we hypothesized that activated T cells that migrate to the bone marrow during the course of EAE may in fact be involved in the mobilization of MSCs. Alternatively, high numbers of activated T cells in bone marrow samples could influence the outcome of the CFU assays through the production of growth factors, which may suppress MSC proliferation or induce MSC apoptosis. To test whether activated CD4^pos^ or CD8^pos^ T cells in general could produce factors which could inhibit the proliferation of MSCs *in vitro*, thereby affecting the outcome of the CFU-F assay, we cultured bone marrow MSCs of healthy mice with supernatants of *in vitro* CD3-CD28 beads activated CD4^pos^ or CD8^pos^ T cells. We labeled MSCs with CFSE and cultured them for 3 days, allowed them to proliferate under normal culture conditions, in the presence or absence of different concentrations of supernatants from activated CD4^pos^ or CD8^pos^ T cells. Upon cell division, CFSE is equally divided over the daughter cells and thus reduced cell division will result in higher MFI levels of the measured CFSE. Although the supernatants contained IFN-y and TNF-α (data not shown), indicating that T cells were indeed activated, we observed no difference in the capacity of MSCs to proliferate upon addition of various concentrations of supernatant derived from activated CD4^pos^ T cells when compared to MSCs cultured under normal conditions (Figure 3A). The same results were obtained with supernatants from activated CD8^pos^ T cells (Figure 3A). Furthermore, culturing CFSE labeled MSCs with different concentrations of various recombinant cytokines, inflammatory molecules, growth factors and potential mobilization factors, revealed that none of these factors substantially inhibited the proliferation of MSCs *in vitro* (Figure 3B). Together, these data indicate that factors produced by activated T cells did not directly affect the proliferation of MSCs and therefore did not influence our CFU assays. Consequently, our data suggest that other mechanisms are involved in the observed decrease in numbers of MSC in the bone marrow during the course of EAE.

To test whether T cells present in the bone marrow produce inflammatory mediators that could instigate the mobilization of MSCs, we determined whether TNF-α, IFN-y, and IL-17 could be detected in supernatants of complete bone marrow samples upon *in vitro* restimulation with rMOG for 72 h. Surprisingly, restimulation did not result in detectable levels of IFN-y or IL-17 (data not shown) and only low levels of TNF-α, which was elevated at day 29 when compared to control bone marrow samples, could be detected (Figure 3C). Since VEGF-A has been shown to be directly involved in mobilization of MSCs from the bone marrow (Pitchford et al., 2009) and CD4^pos^ T cells are able to produce VEGF-A upon activation (Matsuyama et al., 2002), we also determined the levels of VEGF-A in these supernatants. Even though detectable levels of VEGF-A could be measured, we did not observe increased VEGF-A levels in bone marrow samples derived from EAE mice when compared to controls, which could account for the mobilization of MSCs (Figure 3C).

Since we showed that the number of bone marrow MSCs is transiently decreased during the course of EAE, we addressed whether these cells could be found at elevated numbers at other locations in the body. We could detect CFU-F activity only in the meninges of EAE mice, and not in control animals, at day 15 after disease induction, although the numbers of precursors were very low (data not shown). In addition, we determined the numbers of MSCs in SPC, and cervical lymph nodes (cLNs) of mice at the chronic phase of EAE (day 29), however, CFU-F analysis of cLNs as well as SPC did not reveal significant differences (Figure 3D).

Collectively, the observed decrease of MSCs within the bone marrow of diseased animals is paralleled by an increase of T cells within the bone marrow. Since inflammatory mediators could not be identified as the responsible effector molecules, direct cell-cell contact between CD4^pos^ or CD8^pos^ T cells and MSCs within the bone marrow could regulate the number of bone marrow MSCs during the course of EAE.

### IFN-y mediated chronic immune activation depletes MSCs in bone marrow

To address whether activated T cells could indeed affect the presence of MSCs in the bone marrow during chronic immune activation, we analyzed the number of MSCs in the bone marrow of B cell specific CD70 transgenic (CD70TG) mice. These mice have a constitutively activated T cell compartment since transgenic over-expression of CD70 by B cells induces T cells to continuously produce high levels of IFN-y, eventually leading to depletion of B cells from bone marrow, spleen, and lymph nodes (Arens et al., 2001; de Bruin et al., 2010). When these mice are crossed with IFN-y^−/−^ mice, B cell numbers remain normal when compared to IFN-y^−/−^ mice, indicating that constitutive production of IFN-y by T cells induces B cell depletion (Arens et al., 2001). In addition, B cell depletion is also a result of extensive triggering of CD27 expressed on hematopoietic precursors (Nolte et al., 2005).

**Figure 4:**
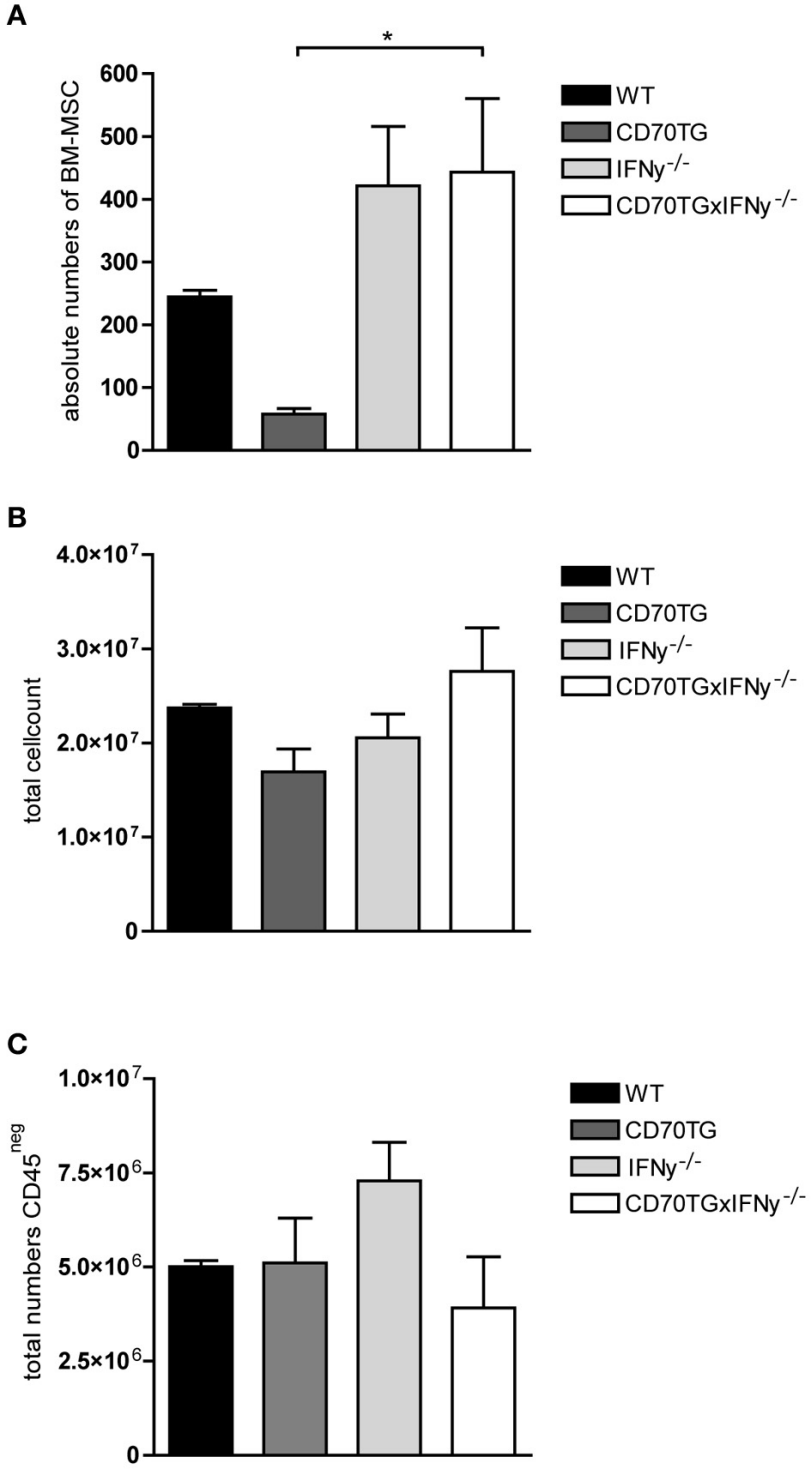
**Chronic immune activation depletes MSCs from the bone marrow. (A)** Absolute numbers of MSCs in bone marrow in WT, CD70TG, and CD70TG/IFN-y^−/−^ mice. **(B)** Total cell count, and **(C)** absolute numbers of CD45^neg^ cells in bone marrow of WT, CD70TG, and CD70TG/IFN-y^−/−^ mice. Data represent mean ± SEM of 3 animals per group (ANOVA; ^*^p < 0.05).

Analysis of the amount of MSCs present in the bone marrow of CD70TG mice revealed a strong reduction in absolute numbers of MSCs when compared to WT controls. Strikingly, this effect was eliminated when CD70TG mice were crossed to IFN-y^−/−^ mice (Figure 4A). It has been reported that CD70TGIFN-y^−/−^ mice still showed an activated T cell compartment as indicated by increased numbers of CD44^hi^CD62L^neg^ T cells in both lymph nodes and bone marrow (Arens et al., 2001; de Bruin et al., 2010), therefore, it is unlikely that the observed decrease in bone marrow MSCs in CD70TG mice is a result of the presence of activated T cells but rather highlights the role of IFN-y produced by T cells in reducing the number of MSCs in the bone marrow. Additionally, the observed differences were neither the result of changes in bone marrow cellularity nor differentiation of MSCs into stromal cells, as we observed no changes in total cell count or the amount of CD45^neg^ cells in CD70TG mice, respectively (Figures 4B,C).

### Mobilization of MSCs depends on T cell derived IFN-y

**Figure 5:**
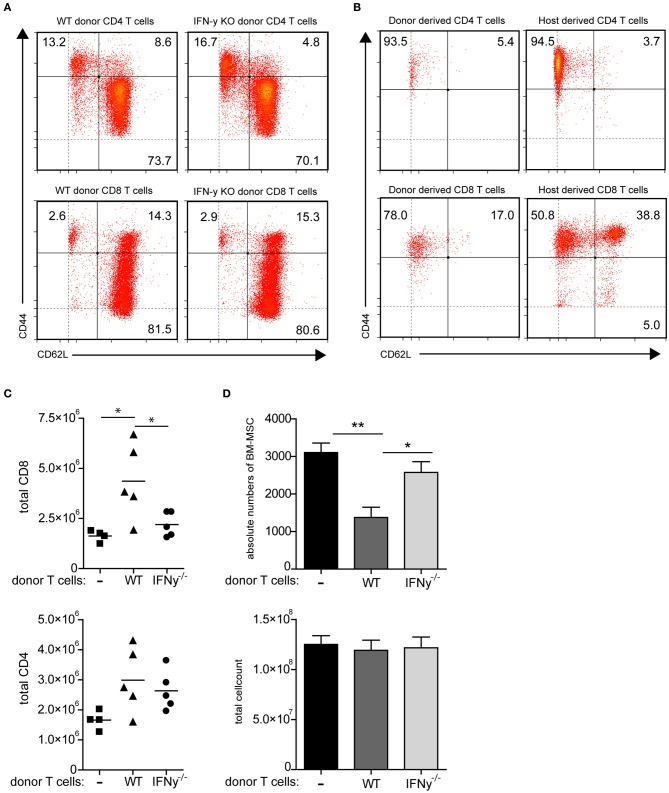
**Mobilization of MSCs depends on T cell derived IFN-y. (A,B)** Phenotype of WT and IFN-y^−/−^ donor CD4 and CD8 T cells before adoptive transfer **(A)** and phenotype of donor WT T cells and host donor T cells in the bone marrow of CD70TGxCD27^−/−^ mice after 3 weeks of adoptive transfer **(B)**, effector memory; CD44^pos^CD62L^neg^, central memory; CD44^pos^CD62L^pos^, naive; CD44^neg^CD62L^pos^. **(C)** Absolute numbers of CD8 or CD4 T cells in bone marrow of CD70TGxCD27^−/−^ recipients that received no T cells, WT T cells or IFN-y^−/−^ T cells. **(D)** Absolute numbers of MSCs and total cell count in bone marrow of CD70TGxCD27^−/−^ recipients that received no T cells, WT T cells or IFN-y^−/−^ T cells. Data represent an average of 5 animals per group (±SEM, ANOVA, ^*^p < 0.05, ^**^p < 0.01).

To more clearly indicate that indeed IFN-y producing activated T cells are responsible for the reduction of MSC in the bone marrow we adoptively transferred WT T cells into CD70TGxCD27^−/−^ mice. Upon transfer, only the donor T cells will become activated through CD27-CD70 interaction, while they subsequently differentiate into effector T cells (Figures 5A,B) and accumulate in the bone marrow. Transfer of WT T cells clearly reduces absolute number of MSCs within 3 weeks when compared to CD70TGxCD27^−/−^ mice (Figure 5D). To assess whether this effect is IFN-y mediated, we also adoptively transferred IFN-y^−/−^ T cells into CD70TGxCD27^−/−^ mice. The transfer of an equal amount of IFN-y^−/−^ T cells was not sufficient to reduce number of MSCs within the same time period (Figure 5D). Transfer of WT T cells or IFN-y^−/−^ T cells did not affect bone marrow cellularity (Figure 5D). Strikingly, we observed increased numbers of CD8 T cells but not CD4 T cells in the bone marrow of CD70TGxCD27^−/−^ that received WT T cells (Figure 5C).

Upon analysis of MSC number after 5 days of transfer of WT T cells or IFN-y^−/−^ T cells we did not observe differences between the two groups (data not shown) suggesting that the reduction of MSCs depends on prolonged T cell activation and IFN-y production.

Together, these data indicate that mobilization of MSC out of the bone marrow is mediated via IFN-y production by activated T cells.

## Discussion

MSCs have emerged as potential therapeutic treatment of MS, since they possess strong immunosuppressive capacity. In EAE, the most commonly used animal model for MS, it was shown that transfer of MSCs before or at the onset of clinical disease, reduced the clinical signs of the disease. By suppressing immune cell activation and thereby the infiltration of immune cells into the CNS, the administration MSCs resulted in reduced demyelination and axonal loss (Zappia et al., 2005; Gerdoni et al., 2007; Kassis et al., 2008; Lanza et al., 2009). Here we addressed whether endogenous MSCs could affect the ongoing disease. MSCs can be found in almost all organs, but they are most frequent in bone marrow (da Silva et al., 2006). We observed a strong reduction in the amount of bone marrow MSCs at the peak of the disease, which returned to control levels during the chronic phase of the disease. We hypothesize that this strong reduction in bone marrow MSCs is due to mobilization of MSCs into the periphery. Analysis of the hematopoietic compartment revealed that high levels of both CD4^pos^ and CD8^pos^ T cells coincided with low numbers of MSCs in the bone marrow. We propose that upon inflammation, activated T cells, which migrate to the bone marrow, can mediate the mobilization of MSCs in an IFN-y dependent way.

Several factors have been proposed to mediate the mobilization of MSCs from the bone marrow. For instance, elevated levels of MSCs are found within 48 h in peripheral blood after experimentally induced femoral vascular injury. The mobilization of MSCs from the bone marrow in this model is possibly mediated by VEGF and G-CSF, since these factors were significantly elevated within 24 h (Wang et al., 2008). Others have shown that combined treatment of mice with VEGF and G-CSF is not sufficient to mobilize MSCs, but that interrupting the SDF1-CXCR4 axis together with VEGF pretreatment is mandatory for MSC mobilization (Pitchford et al., 2009). In both studies, mobilization is not mediated by immune cells and in the latter study, mice were not exposed to tissue injury.

Under normal, non-pathogenic conditions, the bone marrow harbors both memory T cells together with T cells that continuously circulate through the body. Additionally, the bone marrow also functions as a secondary lymphoid organ, since priming of naïve T cells can take place within the bone marrow (Feuerer et al., 2003). Moreover, the bone marrow can actively recruit activated T cells which is mediated through alpha 2-integrin (Di Rosa and Pabst, 2005; Tokoyoda et al., 2009). We hypothesized that activated T cells present in the bone marrow could influence the number of bone marrow MSCs. To test this, we analyzed B cell specific CD70TG mice, which contain T cells that are constitutively activated and produce IFN-y continuously. As a control we used CD70TGxIFN-y^−/−^ mice, which still have an activated T cell compartment, but lack the production of IFN-y (Arens et al., 2001). Whereas in CD70TG mice, numbers of MSCs were severely reduced, this phenomenon was completely abrogated in CD70TG mice that were deficient for IFN-y, demonstrating that the reduced MSC numbers were a result of continuous IFN-y production in the bone marrow, most likely derived from activated T cells. Activated T cells express the adhesion molecule CD44, which serves as a ligand for hyaluronic acid that colocalizes with CXCL12, produced in the bone marrow by both endothelial cells and stromal cells including MSCs (Avigdor et al., 2004). Therefore, during immune activation, T cells could specifically localize close to MSCs, secrete IFN-y locally, thereby mediating MSC mobilization. Since the CD70TGxIFN-y^−/−^ mice still contain an activated T cell compartment, including CD44 expressing activated T cells, it is unlikely that mobilization of MSCs is caused only through physical interaction with activated T cells, but rather suggests that paracrine secretion of IFN-y by T cells is needed. Indeed, CD4^pos^ and CD8^pos^ T cells in the bone marrow of CD70TG mice were shown to produce high levels of IFN-y upon measurement by FACS analysis (Arens et al., 2001). Remarkably, systemic increase of IFN-y levels could not be measured in these mice suggesting that the action of IFN-y on MSCs is mediated when T cells and MSCs are in close proximity. In addition, upon activation, T cells are able to produce VEGF-A (Matsuyama et al., 2002). Whether VEGF-A production by activated T is under the influence of IFN-y and abrogated in CD70TG/IFN-y^−/−^ is unknown but could explain the lack of mobilization in these mice, despite an active T cell compartment.

The observation that adoptive transfer of WT T cells but not IFN-y^−/−^ T cells into CD70TGxCD27^−/−^ resulted in decreased MSC numbers in the bone marrow supports the hypothesis of T cell mediated, IFN-y dependent reduction of MSCs. Shortly after transfer, we were unable to observe differences between CD70TGxCD27^−/−^ mice that received WT T cells or IFN-y^−/−^ T cells indicating that only upon prolonged T cell activation and IFN-y production by donor T cells, MSC numbers are reduced from the bone marrow.

We did not observe increased cell death upon *in vitro* stimulation of MSCs with IFN-y (data not shown), suggesting that MSCs are resistant to IFN-y mediated apoptosis as was observed to occur with B cells. Analysis of complete EAE bone marrow samples upon *in vitro* restimulation with MOG did not reveal increased IFN-y levels, suggesting that either antigen specific T cells are suppressed or completely absent from the bone marrow during EAE, or that IFN-y production by MOG-specific T cells cannot be measured in the supernatants. Decreased effector functions of T cells upon *in vitro* restimulation would be in line with other publications showing that MSCs are able to inhibit T cell activation (Zappia et al., 2005; Gerdoni et al., 2007; Kassis et al., 2008). Therefore, the interaction between IFN-y producing T cells and MSCs will critically control both cell subsets, since suppression of activated IFN-y producing T cells by MSCs may sustain the MSC population in the bone marrow, while loss of this control will ensure their mobilization from the bone marrow.

It is unclear whether bone marrow MSCs that disappear from the bone marrow during the disease, localize to distinct organs. Based on CFU-F assays, we could observe MSCs in the meninges of EAE mice at day 15 (data not shown). Although the numbers of CFUs were very low, these colonies could never be observed in control mice. Several studies indicate that upon systemic administration, MSCs migrate to lymphoid organs as well as to the brain and SPC (Zappia et al., 2005; Kassis et al., 2008). We did not observe significant differences between MSCs present in SPCs of diseased versus non-diseased mice, although this may need further analysis at different timepoints. It could however be that subsequent differentiation of MSCs within the affected tissue prevents their CFU-F capacity in our assays, thus limiting our ability to detect them.

The observation that bone marrow MSCs are mobilized from the bone marrow at the peak of disease could have a similar effect on the course of the disease as the therapeutic administration of exogenous MSCs, which is most effective before or at the peak of disease. These data suggests the need for a temporal systemic increase of MSCs before clinical disease onset to suppress the priming and activation of autoimmune/antigen specific T cells. Future studies that induce the active mobilization and proliferation of endogenous bone marrow MSCs before onset of clinical disease would indicate whether the endogenous MSCs can dampen the effects of T cell mediated autoimmunity.

## Experimental procedures

### Animals

EAE was induced in female FVB mice (NKI, Amsterdam) or C57BL/6 (Charles River, France), 8–12 weeks of age and housed at the VU University Medical Center. B cell-specific CD70TG (Arens et al., 2001), IFN-γ^−/−^, CD70TGxIFN-γ^−/−^, CD70TGxCD27^−/−^, and wild-type control mice were housed and used at the AMC, Amsterdam. All experimental procedures were reviewed and approved by the Ethical Committee for Animal Experiments of the VU (Vrije Universiteit) University Medical Center (Amsterdam, The Netherlands).

### Induction of EAE

EAE was induced in 8–12-week-old female mice via subcutaneous immunization with 200 μg rMOG (1–125; synthesized as described Adelmann et al., 1995) mixed (volume ratio 1:1) with Complete Freund's Adjuvant (CFA; Difco Laboratories) containing 500 μg of heat-killed *Mycobacterium tuberculosis* H37Ra (MBT; Difco). Control (CFA) animals were injected with saline mixed with CFA containing 500 μg of heat-killed MBT. All animals were additionally injected intraperitoneally (i.p.) with 200 ng pertussis toxin derived from *Bordetella pertussis* (Sigma, Zwijndrecht, The Netherlands) in 200 μL saline at the time of, and 24 h after immunization. Mice were examined daily for clinical signs of EAE and were scored as follows: 0, no disease; 1, limp tail; 2, hind limb weakness; 3, complete hind limb paralysis; 4, hind limb paralysis plus fore limb paralysis; and 5, moribund or dead. Mice were euthanized at day 8, 15, or 29.

### Single cell suspension

Bone marrow single cell suspensions were obtained according to the following protocol. Freshly isolated femur and tibia were flushed once with 1 ml icecold DMEM (Gibco) supplemented with 2% FCS, 2% antibiotics, and glutamine *(Flush Fraction)*. To release MSCs from the bone marrow, femur, and tibia were subsequently incubated with DMEM containing Blendzyme 2 (150 μg/ml), DNAse I (200 ug/ml, both Roche Applied Sciences, The Netherlands), 2% FCS, and 2% antibiotics for 20 min at 37°C under continuous stirring. After incubation, enzymatic activity was stopped and femur and tibia were flushed once more with 1 ml cold DMEM with 2% FCS and 2% antibiotics and all cells were collected *(Blendzyme 2 fraction)*.

Lymph node, SPC and meninges single cell suspension were obtained as follows. Isolated cLNs, SPC, and meninges were cut extensively into small pieces followed by enzymatic digestion in DMEM containing Blendzyme 2 (150 μg/ml), DNAse I (200 ug/ml), 2% FCS, and 2% antibiotics for 15 min at 37°C under continuous stirring. After incubation, enzymatic activity was stopped by adding cold DMEM containing 10% FCS and 2% antibiotics.

All single cell suspensions were washed once with an excess of medium to remove any residual Blendzyme 2 and resuspended in DMEM (Gibco) supplemented with 2% FCS, 2% antibiotics, and glutamine, and cells were counted. For flow cytometry analysis, cells were passed through a 70 μm filter to remove cell clumps.

### Adoptive transfer

T cells were obtained from lymph nodes and spleen single cell suspensions from either WT (CD45.1^pos^) or IFN-y^−/−^ mice. Single cell suspensions were generated by mincing the organs through 40 μm cell strainers followed by erythrocyte lysis with ACK lysis buffer. Subsequently, cells were incubated with CD4 and CD8 microbeads (Miltenyi Biotec) and enriched by MACS positive selection using automacs with LS columns (Miltenyi Biotec). Enriched cells with a purity of >95% as determined by flow cytometry, were resuspended in PBS and 5–10 × 10^6^ in 200 μ l PBS cells were intravenously injected into CD70TGxCD27^−/−^ mice. Mice were sacrificed and analyzed 5 days or 21 days after T cell transfer.

### Colony forming unit assay

Colony forming unit (CFU) assays were performed according to the following protocol: single cell suspensions were seeded at three different concentrations in 2 ml Mesencult + stimulatory supplements (Mesencult Proliferation Kit, Stem Cell Technologies, France) and 2% antibiotics/well in a 6-wells plate. CFU assays were always performed in duplo. After 2 weeks of culture, adherent cells were washed twice with PBS, fixed in methanol and stained with Giemsa. CFU's were counted using a stereo microscope. A cluster of cells containing ≥10 cells was considered a colony.

### Flow cytometry

Single cell suspensions were seeded at 2 × 10^6^ cells/well in a 96 well plate (4°C). All cells were incubated with 10% (vol/vol) normal mouse serum (NMS) to block aspecific binding of primary antibodies. Subsequently, staining with the appropriate antibodies was carried out for 30 min at 4°C.

The following antibodies, recognizing CD45R (clone RA3-6B2), CD8 (clone 53-6.7), and CD31 (clone ERMP12) were affinity purified from the supernatants of hybridoma cell cultures by using protein G-sepharose (Pharmacia, Uppsala, Sweden). The antibodies were either biotinylated or labeled with Alexa-Fluor-488 or Alexa-Fluor-647 (Invitrogen Life Technologies). Unless stated otherwise, the following antibodies were obtained from eBioscience; CD8-FITC (clone Ly-2), CD45.1-FITC (BD Biosciences, Clone A20), GR-1-Alexa-Fluor-488 (clone RB6-8C5), CD8-PE (clone Ly-2), CD19-PE (BD Pharmingen clone ID3), CD44-PE (clone IM-7), F4/80-PE (clone BM8), SCA-1 PE-Cy5.5 (clone D7), CD45-PE-Cy7 (clone 30F-11), CD4-APC (clone GK1.5), CD62L-APC (clone MEL-14) IFN-y-APC (clone XMG1.2), CD11c-APC (clone N418), CD45 Pacific Orange (Caltag Laboratories, clone 30-F11), and CD11b-biotinylated (Beckman Coulter, clone M1/70). Biotinylated antibodies were visualized with Streptavidin-APC-Cy7 (eBioscience). Sytox Blue dead cell stain (Molecular Probes) was used to discriminate between live and dead cells. Data were acquired on a Cyan ADP High Performance Research Flow Cytometer (Beckman Coulter) and were analyzed with Summit Software v4.3. Single stained cells were used to compensate for spectral overlap. Fluorescence Minus One (FMO) stained cells were used to set boundaries between positively and negatively stained cells.

### *In vitro* restimulation and luminex assay

Bone marrow single cell suspensions were seeded at 1 × 10^6^ cells/well in a flat-bottomed 96 well plate in media [IMDM supplemented with 10% FCS, 50 μ M β-mercaptoethanol, 2% penicillin, streptomycin, and L-glutamine (PSG)]. Cells were stimulated with either various concentrations (3–30 μg/ml) rMOG (1–125) or PMA (20 ng/ml; Sigma)/ionomycin (500 ng/ml; Sigma) as a positive control. Supernatants were harvested after 72 h and stored in −20°C until further analysis. IFN-y, IL-17, TNF-α, VEGF, and GM-CSF were measured using Luminex assay (Millipore Corporation, USA, cat.nr. mpxmcyto-70k) according to the manufacturer's instructions.

*For in vitro* restimulation of bone marrow T cells, 1 × 10^6^ total bone marrow cells were restimulated for 6 h with PMA (10 ng/ml) and Ionomycin (1 μ M) in the presence of Brefeldin A (Golgiplug™, BD biosciences). The percentage of IFN-y producing T cells was measured with flow cytometry.

### *In vitro* MSC proliferation

Bone marrow single cell suspensions from healthy C57BL/6 mice were obtained as described above. To expand MSCs *in vitro*, bone marrow single cell suspensions were cultured overnight in Mesencult + stimulatory supplements and 2% antibiotics. After 24 h, non-adherent cells were removed and fresh medium was added. After two passages, cells were used for proliferation assays. Hereto, cells were harvested upon trypsinization and washed extensively with PBS. Cells were labeled with 5 μM 5,6-carboxyfluorescein succinimidyl ester (CFSE, Moleculare Probes, Invitrogen) at 1 × 10^7^ cells/ml for 10 min at 37°C. After washing, the cells were seeded at 0.1 × 10^6^ cells per well in a 6-wells plate supplemented with 2 ml Mesencult + stimulatory supplements. Cells were allowed to adhere for 2 h and subsequently incubated with the following stimuli at various concentrations (0.25 ng/ml, 2.5 ng/ml, and 25 ng/ml); IL-1, IL-6, IL-10, IL-17, IFN-y, TNF-α, G-CSF, GM-CSF, VEGF, and TGF-β (all peprotech, UK).

T cell conditioned medium was prepared as follows; spleens were isolated from C57BL/6 mice and minced through 70 μm gauze to obtain single cell suspensions. Red blood cells (RBCs) were removed with lysis buffer (150 mM NH_4_, 1 mM NaHCO_3_, pH 7.4). CD4 or CD8 T cells were labeled with either CD4-PE-Cy7 (clone GK1.5) or CD8-PE-Cy7 (clone 53-6.7) antibodies (both eBioscience) and isolated with the Easysep Mouse PE Positive selection kit (Stem Cell Technologies, France) according to manufacturer's instructions. Purity was checked by flowcytometry (>95% purity). Subsequently, purified T cells were stimulated with CD3/CD28 beads (BD Bioscience) and after 72 h, T cell conditioned supernatants were collected and stored at −20°C. For bone marrow MSC proliferation assay, T cell conditioned medium was diluted 1:2, 1:5, or 1:10 and added to plate adherent MSCs. For all proliferation assays, unstimulated CFSE labeled cells were used as controls. After 72 h, cells were harvested by trypsinization and CFSE dilution was analyzed on a Cyan ADP as a measurement for proliferation. Sytox Blue dead cell staining was used to discriminate between live and dead cells.

### Conflict of interest statement

The authors declare that the research was conducted in the absence of any commercial or financial relationships that could be construed as a potential conflict of interest.

## References

[B1] Adelmann M. Wood J. Benzel I. Fiori P. Lassmann H. Matthieu J. M. (1995). The N-terminal domain of the myelin oligodendrocyte glycoprotein (MOG) induces acute demyelinating experimental autoimmune encephalomyelitis in the Lewis rat. J. Neuroimmunol. 63, 17–27 8557821 10.1016/0165-5728(95)00124-7

[B2] Arens R. Tesselaar K. Baars P. A. van Schijndel G. M. Hendriks J. Pals S. T. (2001). Constitutive CD27/CD70 interaction induces expansion of effector-type T cells and results in IFNgamma-mediated B cell depletion. Immunity 15, 801–812 10.1016/S1074-7613(01)00236-9 11728341

[B3] Avigdor A. Goichberg P. Shivtiel S. Dar A. Peled A. Samira S. (2004). CD44 and hyaluronic acid cooperate with SDF-1 in the trafficking of human CD34+ stem/progenitor cells to bone marrow. Blood 103, 2981–2989 10.1182/blood-2003-10-3611 15070674

[B4] Bruck W. Kuhlmann T. Stadelmann C. (2003). Remyelination in multiple sclerosis. J. Neurol. Sci. 206, 181–185 12559508 10.1016/s0022-510x(02)00191-0

[B5] Codarri L. Gyulveszi G. Tosevski V. Hesske L. Fontana A. Magnenat L. (2011). RORgammat drives production of the cytokine GM-CSF in helper T cells, which is essential for the effector phase of autoimmune neuroinflammation. Nat. Immunol. 12, 560–567 10.1038/ni.2027 21516112

[B6] da Silva M. L. Chagastelles P. C. Nardi N. B. (2006). Mesenchymal stem cells reside in virtually all post-natal organs and tissues. J. Cell Sci. 119, 2204–2213 10.1242/jcs.02932 16684817

[B7] de Bruin A. M. Buitenhuis M. van der Sluijs K. F. van Gisbergen K. P. Boon L. Nolte M. A. (2010). Eosinophil differentiation in the bone marrow is inhibited by T cell-derived IFN-gamma. Blood 116, 2559–2569 10.1182/blood-2009-12-261339 20587787

[B8] Ding L. Saunders T. L. Enikolopov G. Morrison S. J. (2012). Endothelial and perivascular cells maintain haematopoietic stem cells. Nature 481, 457–462 10.1038/nature10783 22281595 PMC3270376

[B9] Di Rosa F. Pabst R. (2005). The bone marrow: a nest for migratory memory T cells. Trends Immunol. 26, 360–366 10.1016/j.it.2005.04.011 15978522

[B10] Eglitis M. A. Dawson D. Park K. W. Mouradian M. M. (1999). Targeting of marrow-derived astrocytes to the ischemic brain. Neuroreport 10, 1289–1292 10363941 10.1097/00001756-199904260-00025

[B11] Ewing C. Bernard C. C. (1998). Insights into the aetiology and pathogenesis of multiple sclerosis. Immunol. Cell Biol. 76, 47–54 10.1046/j.1440-1711.1998.00718.x 9553776

[B12] Fancy S. P. Kotter M. R. Harrington E. P. Huang J. K. Zhao C. Rowitch D. H. (2010). Overcoming remyelination failure in multiple sclerosis and other myelin disorders. Exp. Neurol. 225, 18–23 10.1016/j.expneurol.2009.12.020 20044992

[B13] Feuerer M. Beckhove P. Garbi N. Mahnke Y. Limmer A. Hommel M. (2003). Bone marrow as a priming site for T-cell responses to blood-borne antigen. Nat. Med. 9, 1151–1157 10.1038/nm914 12910264

[B14] Friedenstein A. J. Piatetzky-Shapiro I. I. Petrakova K. V. (1966). Osteogenesis in transplants of bone marrow cells. J. Embryol. Exp. Morphol. 16, 381–390 5336210

[B15] Fry T. J. Mackall C. L. (2005). The many faces of IL-7, from lymphopoiesis to peripheral T cell maintenance. J. Immunol. 174, 6571–6576 15905493 10.4049/jimmunol.174.11.6571

[B16] Gerdoni E. Gallo B. Casazza S. Musio S. Bonanni I. Pedemonte E. (2007). Mesenchymal stem cells effectively modulate pathogenic immune response in experimental autoimmune encephalomyelitis. Ann. Neurol. 61, 219–227 10.1002/ana.21076 17387730

[B17] Hemmer B. Archelos J. J. Hartung H. P. (2002). New concepts in the immunopathogenesis of multiple sclerosis. Nat. Rev. Neurosci. 3, 291–301 10.1038/nrn784 11967559

[B18] Jones S. Horwood N. Cope A. Dazzi F. (2007). The antiproliferative effect of mesenchymal stem cells is a fundamental property shared by all stromal cells. J. Immunol. 179, 2824–2831 17709496 10.4049/jimmunol.179.5.2824

[B19] Kassis I. Grigoriadis N. Gowda-Kurkalli B. Mizrachi-Kol R. Ben-Hur T. Slavin S. (2008). Neuroprotection and immunomodulation with mesenchymal stem cells in chronic experimental autoimmune encephalomyelitis. Arch. Neurol. 65, 753–761 10.1001/archneur.65.6.753 18541795

[B20] Koning J. J. Mebius R. E. (2011). Interdependence of stromal and immune cells for lymph node function. Trends Immunol. 33, 264–270 10.1016/j.it.2011.10.006 22153930

[B21] Kooij G. Backer R. Koning J. J. Reijerkerk A. van H. J. van der Pol S. M. (2009). P-glycoprotein acts as an immunomodulator during neuroinflammation. PLoS ONE 4:e8212 10.1371/journal.pone.0008212 19997559 PMC2785479

[B22] Kopen G. C. Prockop D. J. Phinney D. G. (1999). Marrow stromal cells migrate throughout forebrain and cerebellum, and they differentiate into astrocytes after injection into neonatal mouse brains. Proc. Natl. Acad. Sci. U.S.A. 96, 10711–10716 10.1073/pnas.96.19.10711 10485891 PMC17948

[B23] Kuznetsov S. A. Krebsbach P. H. Satomura K. Kerr J. Riminucci M. Benayahu D. (1997). Single-colony derived strains of human marrow stromal fibroblasts form bone after transplantation *in vivo*. J. Bone Miner. Res. 12, 1335–1347 10.1359/jbmr.1997.12.9.1335 9286749

[B24] Kuznetsov S. A. Mankani M. H. Gronthos S. Satomura K. Bianco P. Robey P. G. (2001). Circulating skeletal stem cells. J. Cell Biol. 153, 1133–1140 10.1083/jcb.153.5.1133 11381097 PMC2174322

[B25] Lanza C. Morando S. Voci A. Canesi L. Principato M. C. Serpero L. D. (2009). Neuroprotective mesenchymal stem cells are endowed with a potent antioxidant effect *in vivo*. J. Neurochem. 110, 1674–1684 10.1111/j.1471-4159.2009.06268.x 19619133

[B26] Lassmann H. Bruck W. Lucchinetti C. (2001). Heterogeneity of multiple sclerosis pathogenesis: implications for diagnosis and therapy. Trends Mol. Med. 7, 115–121 10.1016/S1471-4914(00)01909-2 11286782

[B27] Matsuyama W. Kubota R. Hashiguchi T. Momi H. Kawabata M. Nakagawa M. (2002). Purified protein derivative of tuberculin upregulates the expression of vascular endothelial growth factor in T lymphocytes *in vitro*. Immunology 106, 96–101 10.1046/j.1365-2567.2002.01395.x 11972637 PMC1782692

[B28] Mendez-Ferrer S. Michurina T. V. Ferraro F. Mazloom A. R. Macarthur B. D. Lira S. A. (2010). Mesenchymal and haematopoietic stem cells form a unique bone marrow niche. Nature 466, 829–834 10.1038/nature09262 20703299 PMC3146551

[B29] Nolte M. A. Arens R. van O. R. van O. M. Hooibrink B. van Lier R. A. (2005). Immune activation modulates hematopoiesis through interactions between CD27 and CD70. Nat. Immunol. 6, 412–418 10.1038/ni1174 15723067

[B30] Noseworthy J. H. Lucchinetti C. Rodriguez M. Weinshenker B. G. (2000). Multiple sclerosis. N. Engl. J. Med. 343, 938–952 10.1056/NEJM200009283431307 11006371

[B31] Pitchford S. C. Furze R. C. Jones C. P. Wengner A. M. Rankin S. M. (2009). Differential mobilization of subsets of progenitor cells from the bone marrow. Cell Stem Cell 4, 62–72 10.1016/j.stem.2008.10.017 19128793

[B32] Pittenger M. F. Mackay A. M. Beck S. C. Jaiswal R. K. Douglas R. Mosca J. D. (1999). Multilineage potential of adult human mesenchymal stem cells. Science 284, 143–147 10.1126/science.284.5411.143 10102814

[B33] Prockop D. J. (1997). Marrow stromal cells as stem cells for nonhematopoietic tissues. Science 276, 71–74 10.1126/science.276.5309.71 9082988

[B34] Scolding N. Franklin R. (1998). Axon loss in multiple sclerosis. Lancet 352, 340–341 10.1016/S0140-6736(05)60463-1 9717917

[B35] Segal B. M. (2010). Th17 cells in autoimmune demyelinating disease. Semin. Immunopathol. 32, 71–77 10.1007/s00281-009-0186-z 20195867 PMC2874248

[B36] Sospedra M. Martin R. (2005). Immunology of multiple sclerosis. Annu. Rev. Immunol. 23, 683–747 10.1146/annurev.immunol.23.021704.115707 15771584

[B37] Steinman L. (2001). Multiple sclerosis: a two-stage disease. Nat. Immunol. 2, 762–764 10.1038/ni0901-762 11526378

[B38] Tokoyoda K. Hauser A. E. Nakayama T. Radbruch A. (2010). Organization of immunological memory by bone marrow stroma. Nat. Rev. Immunol. 10, 193–200 10.1038/nri2727 20154734

[B39] Tokoyoda K. Zehentmeier S. Hegazy A. N. Albrecht I. Grun J. R. Lohning M. (2009). Professional memory CD4+ T lymphocytes preferentially reside and rest in the bone marrow. Immunity 30, 721–730 10.1016/j.immuni.2009.03.015 19427242

[B40] Uccelli A. Moretta L. Pistoia V. (2008). Mesenchymal stem cells in health and disease. Nat. Rev. Immunol. 8, 726–736 10.1038/nri2395 19172693

[B41] Wang C. H. Cherng W. J. Yang N. I. Kuo L. T. Hsu C. M. Yeh H. I. (2008). Late-outgrowth endothelial cells attenuate intimal hyperplasia contributed by mesenchymal stem cells after vascular injury. Arterioscler. Thromb. Vasc. Biol. 28, 54–60 10.1161/ATVBAHA.107.147256 17991877

[B42] Zamvil S. S. Steinman L. (1990). The T lymphocyte in experimental allergic encephalomyelitis. Annu. Rev. Immunol. 8, 579–621 10.1146/annurev.iy.08.040190.003051 2188675

[B43] Zappia E. Casazza S. Pedemonte E. Benvenuto F. Bonanni I. Gerdoni E. (2005). Mesenchymal stem cells ameliorate experimental autoimmune encephalomyelitis inducing T-cell anergy. Blood 106, 1755–1761 10.1182/blood-2005-04-1496 15905186

